# Red Quinoa Bran Extracts Protects against Carbon Tetrachloride-Induced Liver Injury and Fibrosis in Mice via Activation of Antioxidative Enzyme Systems and Blocking TGF-β1 Pathway

**DOI:** 10.3390/nu11020395

**Published:** 2019-02-13

**Authors:** Ting-An Lin, Bo-Jun Ke, Cheng-Shih Cheng, Jyh-Jye Wang, Bai-Luh Wei, Chun-Lin Lee

**Affiliations:** 1 Department of Life Science, National Taitung University, Taitung 950, Taiwan; tingan81@gmail.com (T.-A.L.); kebojun@hotmail.com (B.-J.K.); blwei@nttu.edu.tw (B.-L.W.); 2 Sinfong Agricultural Technology Company, Taipei 11267, Taiwan; wacau88@yahoo.com.tw; 3 Department of Nutrition and Health Science, Fooyin University, Kaohsiung 831, Taiwan; FT054@fy.edu.tw

**Keywords:** *Chenopodium formosanum* Koidz, red quinoa, rutin, liver injury, liver fibrosis

## Abstract

The late stages of liver fibrosis are considered to be irreversible. Red quinoa (*Chenopodium formosanum* Koidz), a traditional food for Taiwanese aborigines, was gradually developed as a novel supplemental food due to high dietary fibre and polyphenolic compounds. Its bran was usually regarded as the agricultural waste, but it contained a high concentration of rutin known as an antioxidant and anti-inflammatory agent. This study is to explore the effect of red quinoa bran extracts on the prevention of carbon tetrachloride (CCl_4_)-induced liver fibrosis. BALB/c mice were intraperitoneally injected CCl_4_ to induce liver fibrosis and treated with red quinoa whole seed powder, bran ethanol extracts, bran water extracts, and rutin. In the results, red quinoa powder provided more protection than rutin against CCl_4_-induced oxidative stress, pro-inflammatory factor expression and fibrosis development. However, the bran ethanol extract with high rutin content provided the most liver protection and anti-fibrosis effect via blocking the tumor necrosis factor alpha (TNF-α)/interleukin 6 (IL-6) pathway and transforming growth factor beta 1 (TGF-β1) pathway.

## 1. Introduction

Liver injury is caused by hepatic virus infection, alcohol abuse, medicine, toxin, abnormal food consumption, metabolic syndrome and other factors. Chronic liver injury can result in liver fibrosis, cirrhosis, liver malfunction and hepatocellular carcinoma [1]. Damaged hepatocytes activate and recruit T cells, which secretes pro-inflammatory cytokines, such as interleukin 6 (IL-6) [2]. A repeated liver injury and wound healing response will lead to liver fibrosis and cirrhosis. Kupffer cells, T cells and macrophages secrete tumour necrosis factor alpha (TNF-α), IL-6 and transforming growth factor beta 1 (TGF-β1). They are important inflammatory mediators. TGF-β1 also mediates activation of hepatic satellites cells (HSCs), which transforms the tissue-resident fibroblasts into activated myofibroblasts. Myofibroblasts produce and remodel connective tissue via an extracellular matrix (ECM) [3]. Currently, no treatment can cure fibrosis and cirrhosis in the liver. However, treatment can delay further liver injury and prevent further ECM accumulation. However, previous studies showed that the early stages of liver fibrosis are reversible. ECM can be degraded by matrix metalloproteinases [4]. Carbon Tetrachloride (CCl_4_) has been widely used to study liver injury and liver fibrosis in animal models. CCl_4_ is converted to trichloromethyl radical in the body mainly by CYP2E1 in the liver. Trichloromethyl radical and oxygen can merge into trichloromethyl peroxyl radical. Those free radicals damage the liver by inducing oxidative stress, lipid peroxidation and triglyceride accumulation in the hepatocytesm [5]. 

Quinoa is known as a superfood. Red quinoa (*Chenopodium formosanum* Koidz) is a native plant in Taiwan. Taiwanese aborigines have harvested since a long time ago for making alcohol. Red quinoa is high in protein (14%), dietary fibre (14%), vitamins, minerals and essential amino acids. It is also high in betacyanins, betaxanthins and flavonoids. Red quinoa has been reported to have anti-oxidation [6] and anti-inflammatory properties, and provide skin protection [7] and hepatic protection. Rutin (vitamin P), a member of flavonoids, is the main bioactive compound in red quinoa [8]. Previous studies have shown that rutin has anti-inflammatory [9], anti-oxidation [10], and anti-tumour qualities, prevents allergic rhinitis [11] and protects the liver [12]. 

In this study, the bran of red quinoa was regarded as the agricultural waste, and was usually removed during the food process. However, the bran of red quinoa contained a high concentration of rutin. Therefore, it can be developed as a functional food for liver protection. In this study, red quinoa bran was extracted with water and ethanol in order to develop a functional food with a high rutin content. Furthermore, this study investigated and compared the effects of red quinoa whole seed powder, bran ethanol extracts, bran water extracts, and rutin on the prevention and mechanism against CCl_4_-induced liver injury and fibrosis in vivo.

## 2. Materials and Methods 

### 2.1. Preparation of Red Quinoa and its Extracts. 

Red quinoa (*Chenopodium formosanum* Koidz) whole seed and bran were obtained from Sin-Fong Farm (Taipei, Taiwan). Red quinoa bran was extracted by five volumes of 50% ethanol at 50 °C for 2 hours. The ethanol extracts were concentrated by using a rotary evaporator and then lyophilized to remove water. Regarding preparation of the red quinoa bran-water extracts, red quinoa bran was extracted using 10 volumes of ultra-pure water at 50 °C for 2 h, concentrated by using a rotary evaporator and then lyophilized to remove water. The concentration of rutin in red quinoa whole seed, red quinoa bran-ethanol extracts, and red quinoa bran-water extracts were determined by using high performance liquid chromatography (HPLC) with a reverse-phase column (Mightysil RP-18 GP 5 μm C18, 250-4.6 mm, Kanto Chemical Co., Inc., Tokyo, Japan) and a diode array detector (DAD, L-2000 series, Hitachi, Japan). The mobile phase (A solvent: 0.1% trifluoroacetic acid; B solvent: acetonitrile) was eluted with 1.0 mL/min of flow rate. Absorption spectra of eluted compounds were recorded at 250 nm. Red quinoa whole seed contained 1.65 mg/g rutin. Red quinoa bran-ethanol extracts contained 12.67 mg/g rutin. Red quinoa bran-water extracts contained 4.68 mg/g rutin.

### 2.2. Animals Grouping and Treatment 

Forty-eight male BALB/c mice were purchased from the National Laboratory Animal Centre (Taipei, Taiwan). They were housed at 23 °C under a 12-h light/dark cycle with a maintained relative humidity of 60% with free access to regular rodent feed and water. After the adaptation period, the animals were weighed and randomly assigned to six groups. The NOR group was administrated corn oil two times weekly (Thursday and Sunday) for 6 weeks via intraperitonea (i.p.) injection and given water orally. The rest of the groups were administrated 0.5 mL/kg BW of CCl_4_ (dissolved in corn oil) two times weekly (Thursday and Sunday) for 6 weeks via IP injection. The CCL group was the CCl_4_ induced liver injury group and given water orally. The HL-P group was fed 5.13 g/kg of red quinoa whole seed powder (rutin 8.46 mg/kg/day) orally. The HL-E group was fed 1.54 g/kg of red quinoa bran-ethanol extracts (rutin 16.4 mg/kg/day) orally. The HL-W group was fed 1.54 g/kg of red quinoa bran-water extracts (rutin 3.92 mg/kg/day) orally. The Rutin group was daily fed 16.4 mg/kg of rutin orally. This animal experiment was reviewed and approved by the Institutional Animal Care and Use Committee (IACUC) of the National Taitung University.

### 2.3. Tissue Sampling

Euthanasia of mice was done via carbon dioxide inhalation. The blood samples were taken from the hepatic portal vein and then stood at room temperature for an hour. The serum was collected by centrifugation at 2000× *g* for 10 minutes at 4 °C and stored immediately at 4 °C before analysis. Liver tissue samples were collected, rinsed by saline solution and immediately frozen in liquid nitrogen, and then stored at −80 °C. The second biggest part of liver tissue samples were fixed in 10% neutral buffered formalin for histology. 

### 2.4. Liver Function Tests and Kidney Function Tests

The alanine transaminase (ALT), aspartate aminotransferase (AST), alkaline phosphatase (ALP), total bilirubin and albumin tests were used to check liver function. The blood urea nitrogen (BUN) and serum creatinine (CRE) tests were used to check kidney function. They were determined by using a chemistry analyser (Beckman-700, Fullerton, CA, USA) with commercial enzymatic kits.

### 2.5. Determination of Thiobarbituric Acid Reactive Substances (TBARS) and Reactive Oxygen Species (ROS) Levels in Hippocampus and Cortex

Hippocampus and cortex homogenates were centrifuged at 4000× *g* for 15 min and the supernatant was used for neurochemical assay. The malondialdehyde (MDA) level was determined using the method of thiobarbituric acid (TBA) colorimetric analysis, and the optical density (OD) value was measured at 532 nm [13]. In the measurement of ROS, homogenates were added to 96-well plates, and nitro blue tetrazolium (NBT) reduction was measured by absorbance at 550 nm in triplicate [14].

### 2.6. Activities of Anti-Oxidative Enzymes

The liver tissue samples were homogenized in phosphate-buffered saline (PBS) buffer and then centrifuged for 15 min at 15,000× *g* to obtain the liver homogenates. The levels of superoxide dismutase (SOD), catalase (CAT) and glutathione peroxidase (GPx) activities were measured using commercial kits (Ransod, SD125, Randox, Crumlin, Antrim, U.K.; ECAT-100, BioAssay Systems, Hayward, CA, USA; RANSEL, RS505, Randox, Crumlin, Antrim, U.K.). The level of glutathione was determined using a EnzyChrom^TM^ GSH/GSSG assay kit (EGTT-100, BioAssay Systems, Hayward, CA, USA).

### 2.7. Histologic Analysis

Liver tissue samples were fixed in 10% neutral buffered formalin, embedded in paraffin and then cut into 7 μm tissue sections. Sections were stained with hematoxylin and eosin (H & E) and with picro-sirius red.

### 2.8. Western Blotting

The liver tissue samples were homogenized in lysis buffer (1% Triton X-100, 20 mM Tris, 40 mM NaF, 0.2% SDS, 0.5% deoxycholate, 1 mM EDTA, 1 mM EGTA, 1 mM Na3VO4, 100 mM NaCl, pH 7.5) and then centrifuged for 15 minutes at 15,000× *g* to obtain the liver homogenates. β-actin (anti-β-actin polyclonal antibodies, ABT264, Millipore, Temecula, CA, USA) and α-SMA (rabbit anti-αSMA monoclonal antibodies, Millipore, Temecula, CA, USA) antibodies were used for Western blotting. 

### 2.9. Enzyme-Linked Immunosorbent Assay

The liver tissue samples were homogenized in ice-cold PBS buffer and then centrifuged for 15 min at 15,000× *g* to obtain the liver homogenates. A self-coating enzyme-linked immunosorbent assay (ELISA) kit (DY008, R & D Systems, Inc., Minneapolis, MN, USA) was used to determine TNF-α and IL-6 levels. Millipore Rabbit anti-TNF-α polyclonal antibodies, Santa Cruz Mouse anti-IL-6 monoclonal antibodies, Thermo goat anti-rabbit IgG with peroxidase conjugated antibodies and Thermo goat anti-mouse IgG with peroxidase conjugated antibodies were used for the ELISA assays. TNF-α and IL-6 recombinant proteins were used as the standard (all from SinoBiological Inc. North Wales, PA, USA). TGF-β1 was determined using a commercial ELISA kit (E-EL-M0051, Elabscience Biotechnology Co., Ltd, Bethesda, MD, USA). 

### 2.10. Statistical Analysis

The data is presented as the mean ± SD. The means followed by a different letter have significant difference (*p* < 0.05). The results were analysed using a one-way ANOVA with Duncan’s multiple rang test. The statistical analyses were performed with IBM SPSS Statistics 12.0 (SPSS Institute, Inc., Chicago, IL, USA).

## 3. Results

### 3.1. Body Weight, Liver Weight and Liver Weight/Body Weight Ratio

The initial body weight in all of groups showed no significant difference (*p* > 0.05). After the second week, the CCL, HL-P, HL-E, HL-W, and Rutin groups had significantly lower body weight than the NOR group (*p* < 0.05). This result showed that CCl_4_ can cause weight loss (as shown in Table 1). Liver injury may cause liver enlargement. In Table 2, the CCL group had significantly higher liver weight than the NOR group (*p* < 0.05) but the CCL group had significantly lower final body weight than the NOR group (*p* < 0.05). This result indicated that the relative liver to body weight in the CCL group was significantly higher than in the NOR group (*p* < 0.05). After the samples’ treatment, the HL-P and Rutin group had significantly lower relative liver to body weight compared to the CCL group (*p* < 0.05). The HL-E and HL-W group had significantly higher relative liver to body weight compared to the CCL group (*p* < 0.05).

### 3.2. The Parameters of Hepatic Function

The AST, ALT, ALP, albumin and bilirubin tests were used to check liver function. According to the results (Table 3), the activities of AST and ALT in the CCL group were significantly higher than in the NOR group by 3.6-fold and 7.5-fold, respectively (*p* < 0.05). After administration of the samples, the HL-P, HL-E, HL-W and Rutin groups had significantly lower levels of AST and ALT activities compared to the CCL group (*p* < 0.05). The groups with CCl_4_ i.p. injection (the CCL, HL-P, HL-E, HL-W and Rutin group) had significantly lower level of ALP activity than the NOR group (*p* < 0.05). The results suggested that the bran ethanol and water extracts both performed significant liver protection, which was as strong as the whole red quinoa powder.

In Table 4, higher levels of the albumin/globulin ratio (A/G ratio) and total bilirubin (TBIL) indicate liver injury. The CCL group had significantly higher levels of total protein, albumin, globulin, A/G ratio and TBIL compared to the NOR group (*p* < 0.05). After all of the samples were treated, the levels of total protein, albumin, globulin, A/G ratio and TBIL decreased significantly (*p* < 0.05); however, the HL-P and HL-E groups were more effective in reducing the TBIL level than the HL-W and Rutin groups.

### 3.3. The Parameters of Renal Function

CCl_4_ i.p. injection not only caused the liver injury but also the renal damage. Therefore, the BUN and serum CRE tests were used to check kidney function in this study. The results (as shown in Table 5) showed that the CCL group had significantly higher levels of BUN and CRE compared to the NOR group (*p* < 0.05). The HL-P and HL-E group had a lower level of BUN than the CCL group (*p* < 0.05). The HL-P, HL-E, HL-W and Rutin groups showed a lower level of CRE than the CCL group (*p* < 0.05).

### 3.4. Liver TBARS Assay

**Figure 1:**
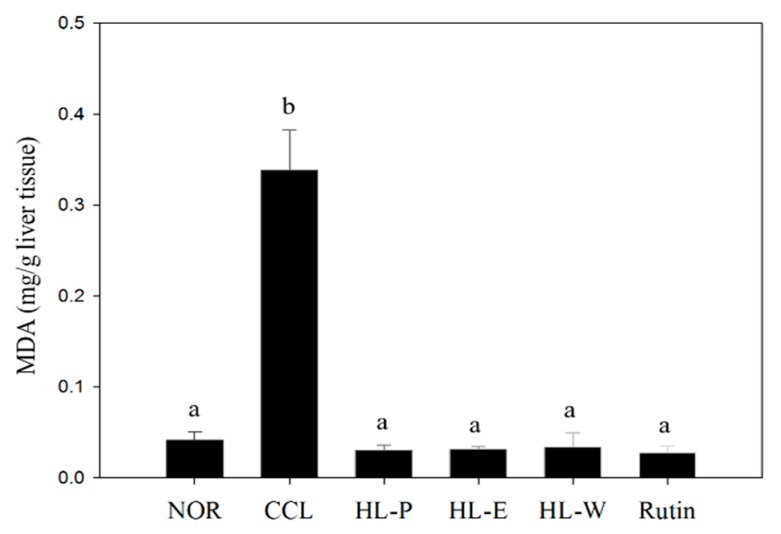
The effects of red quinoa and its extracts on the liver TBARS assay of CCl_4_-induced liver injury mice. NOR: normal group, CCL: CCl_4_-induced liver injury mice, HL-P: CCl_4_-induced liver injury mice fed 5.13 g/kg (rutin 8.46 mg/kg) of red quinoa-powder, HL-E: CCl_4_-induced liver injury mice fed 1.54 g/kg (rutin 16.4 mg/kg) of red quinoa-ethanol extracts, HL-W: CCl_4_-induced liver injury mice fed 1.54 g/kg (rutin 3.92 mg/kg) of red quinoa-water extracts, Rutin: CCl_4_-induced liver injury mice fed 16.4 mg/kg of rutin. Data are presented as mean ± SD (*n* = 8). ^a,b^ Different letters indicate significantly different values according to a one-way ANOVA using Duncan’s multiple test (*p* < 0.05).

CCl_4_ metabolism generates free radicals and causes oxidative stress in liver. The TBARS assay was used to determine lipid peroxidation in this study. In Figure 1, the CCL group had significantly higher levels of MDA in the liver, compared to the NOR group (*p* < 0.05). After administration of the samples, MDA levels in all of the sample groups were significantly decreased (*p* < 0.05). Therefore, the oxidative stress may be repressed by a raised antioxidative system mediated by red quinoa and its bran extract. Anti-oxidative enzyme activity should be further investigated.

### 3.5. The Activities of Anti-Oxidative Enzymes in Liver

**Figure 2:**
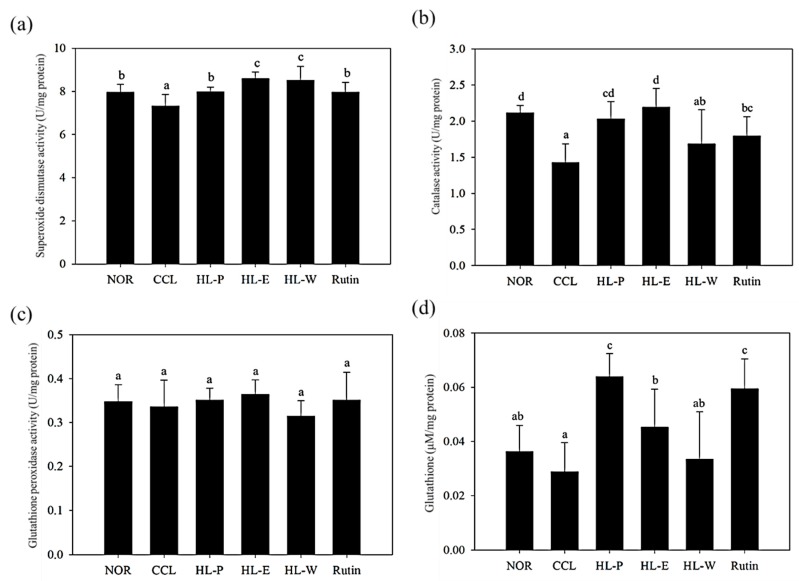
The effects of red quinoa and its extracts on the activities of superoxide dismutase (**a**), catalase (**b**) and glutathione peroxidase (**c**), and the level of glutathione (**d**) of CCl_4_-induced liver injury mice. NOR: normal group, CCL: CCl_4_-induced liver injury mice, HL-P: CCl_4_-induced liver injury mice fed 5.13 g/kg (rutin 8.46 mg/kg) of red quinoa-powder, HL-E: CCl_4_-induced liver injury mice fed 1.54 g/kg (rutin 16.4 mg/kg) of red quinoa-ethanol extracts, HL-W: CCl_4_-induced liver injury mice fed 1.54 g/kg (rutin 3.92 mg/kg) of red quinoa-water extracts, Rutin: CCl_4_-induced liver injury mice fed 16.4 mg/kg of rutin. Data are presented as mean ± SD (*n* = 8). ^a,b,c,d^ Different letters indicate significantly different values according to a one-way ANOVA using Duncan’s multiple test (*p* < 0.05).

Anti-oxidative enzymes can convert free radicals and reactive oxygen species (ROS) into water and oxygen. The levels of SOD and CAT activities in the CCL group were significantly decreased by CCl_4_ i.p. injection (*p* < 0.05) (as shown in Figure 2A,B). After administration of the samples, the levels of SOD and CAT activities in all of the sample groups were significantly increased (*p* < 0.05) compared to the CCL group. In Figure 2C, the level of GPx in the CCL group showed no difference relative to the NOR group and all of the sample groups (*p* > 0.05). GSH is an anti-oxidative agent. The CCL group in GSH level showed no significant difference to the NOR and HL-W groups (*p* > 0.05). However, the HL-P, HL-E and Rutin groups had a significantly higher level of GSH compared to the NOR group (*p* < 0.05) (as shown in Figure 2D). Therefore, the results proved that bran ethanol extract or whole crop powder including rich rutin contents not only activated the anti-oxidative enzymes system but also the GSH levels for the liver protection against CCl_4_-induced oxidative stress.

### 3.6. Hepatic Pathological Changes and Collagen Accumulation

**Figure 3:**
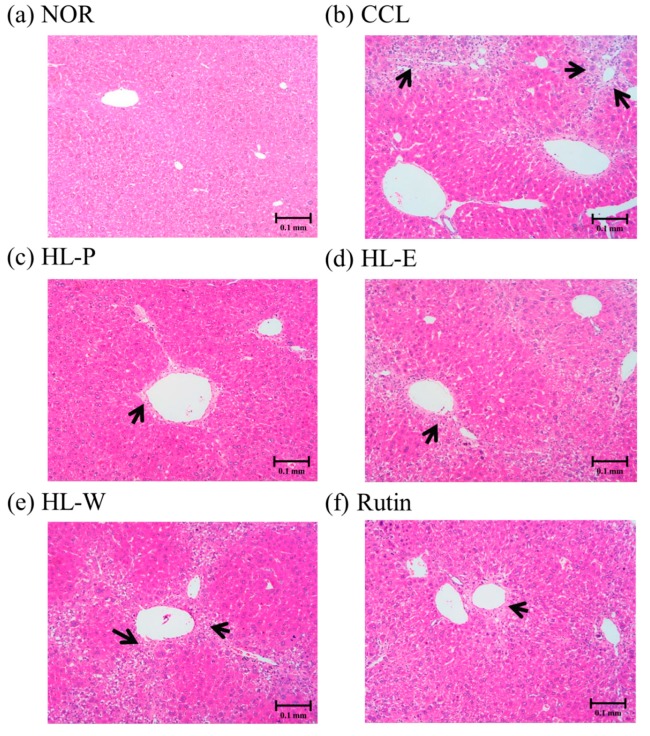
The effects of red quinoa and its extracts on hepatic pathological changes of CCl_4_-induced liver injury mice. Magnification 100× (black arrows indicate the position of liver injury). (**a**) NOR: normal group. (**b**) CCL: CCl_4_-induced liver injury mice. (**c**) HL-P: CCl_4_-induced liver injury mice fed 5.13 g/kg (rutin 8.46 mg/kg) of red quinoa-powder. (**d**) HL-E: CCl_4_-induced liver injury mice fed 1.54 g/kg (rutin 16.4 mg/kg) of red quinoa-ethanol extracts. (**e**) HL-W: CCl_4_-induced liver injury mice fed 1.54 g/kg (rutin 3.92 mg/kg) of red quinoa-water extracts. (**f**) Rutin: CCl_4_-induced liver injury mice fed 16.4 mg/kg of rutin.

**Figure 4:**
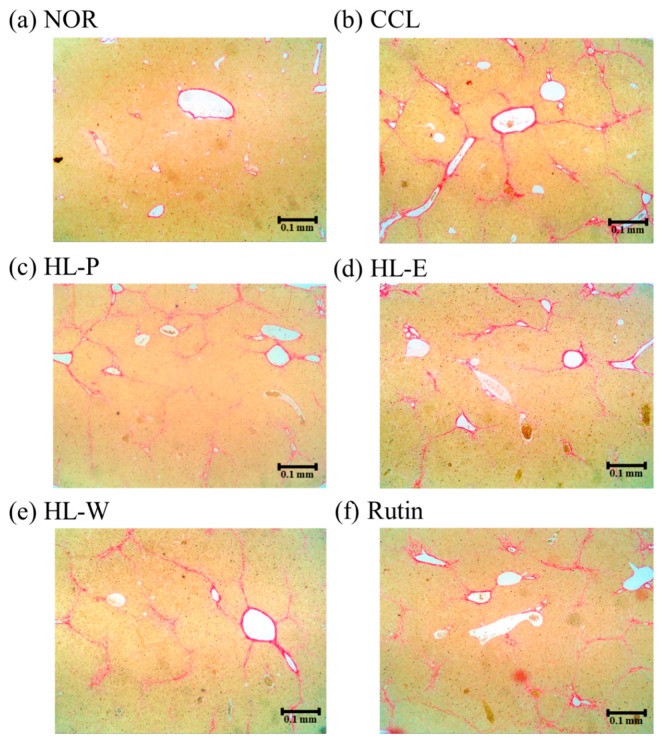
The effects of red quinoa and its extracts on hepatic collagen staining of CCl_4_-induced liver injury mice (Sirius red stain, 40×). (**a**) NOR: normal group. (**b**) CCL: CCl_4_-induced liver injury mice. (**c**) HL-P: CCl_4_-induced liver injury mice fed 5.13 g/kg (rutin 8.46 mg/kg) of red quinoa-powder. (**d**) HL-E: CCl_4_-induced liver injury mice fed 1.54 g/kg (rutin 16.4 mg/kg) of red quinoa-ethanol extracts. (**e**) HL-W: CCl_4_-induced liver injury mice fed 1.54 g/kg (rutin 3.92 mg/kg) of red quinoa-water extracts. (**f**) Rutin: CCl_4_-induced liver injury mice fed 16.4 mg/kg of rutin.

H&E staining of liver tissue in the CCL group showed that CCl_4_ i.p. injection caused severe inflammation, granulomatous calcification and necrosis in the liver. After administration of the samples, the HL-P, HL-E, HL-W and Rutin group had sight inflammation and macrophage infiltration (Figure 3). In the picro-sirius red staining histology tissue images (Figure 4), the CCL group had server collagen accumulation in the liver, indicating the CCl_4_ i.p. injection had caused the fibrosis in the liver. However, the collagen accumulation was inhibited in the HL-P, HL-E, HL-W and Rutin groups.

### 3.7. Pro-Inflammatory and Fibrosis Factors Expression

**Figure 5:**
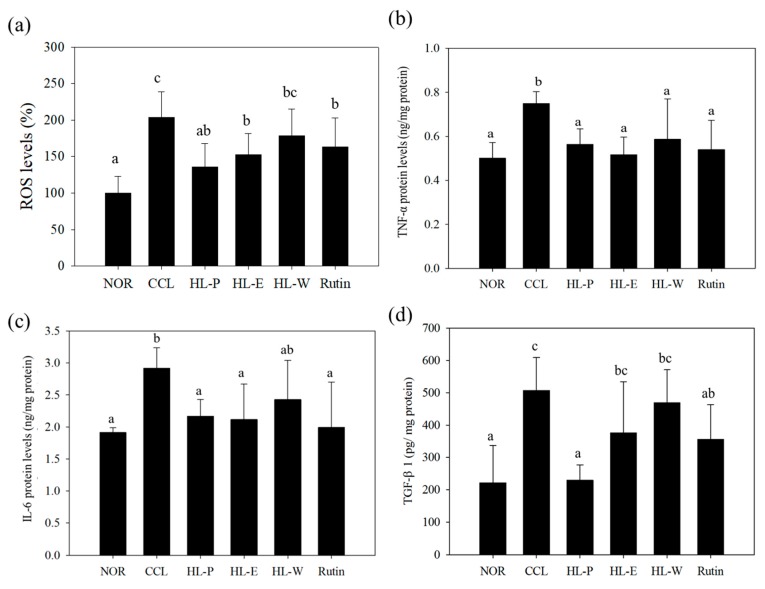
The effects of red quinoa and its extracts on ROS (**a**), TNF-α (**b**), IL-6 (**c**), and TGF-β1 (**d**) of CCl_4_-induced liver injury mice. TNF-α and IL-6 protein levels were measured using an indirect ELISA method. NOR: normal group, CCL: CCl_4_-induced liver injury mice, HL-P: CCl_4_-induced liver injury mice fed 5.13 g/kg (rutin 8.46 mg/kg) of red quinoa-powder, HL-E: CCl_4_-induced liver injury mice fed 1.54 g/kg (rutin 16.4 mg/kg) of red quinoa-ethanol extracts, HL-W: CCl_4_-induced liver injury mice fed 1.54 g/kg (rutin 3.92 mg/kg) of red quinoa-water extracts, Rutin: CCl_4_-induced liver injury mice fed 16.4 mg/kg of rutin. Data are presented as mean ± SD (*n* = 6). ^a,b,c^ Different letters indicate significantly different values according to a one-way ANOVA using Duncan’s multiple test (*p* < 0.05).

**Figure 6:**
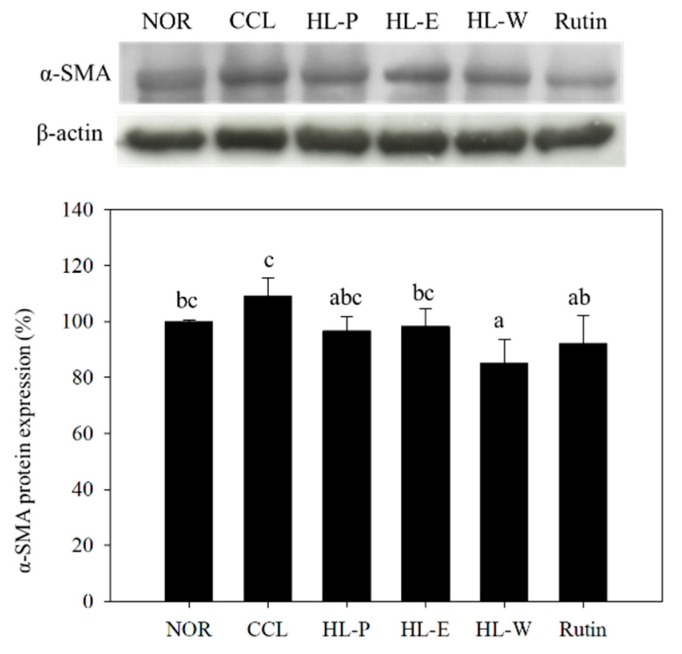
The effects of red quinoa and its extracts on α-SMA of CCl_4_-induced liver injury mice. NOR: normal group, CCL: CCl_4_-induced liver injury mice, HL-P: CCl_4_-induced liver injury mice fed 5.13 g/kg (rutin 8.46 mg/kg) of red quinoa-powder, HL-E: CCl_4_-induced liver injury mice fed 1.54 g/kg (rutin 16.4 mg/kg) of red quinoa-ethanol extracts, HL-W: CCl_4_-induced liver injury mice fed 1.54 g/kg (rutin 3.92 mg/kg) of red quinoa-water extracts, Rutin: CCl_4_-induced liver injury mice fed 16.4 mg/kg of rutin. Data are presented as mean ± SD (n = 8). ^a,b,c,^ Different letters indicate significantly different values according to a one-way ANOVA using Duncan’s multiple test (*p* < 0.05).

The inflammatory response was regarded as the important risk for the development of liver injury because ROS would cause serious liver damage. In addition, the proinflammatory factor TNF-α promoted the expression of TGF-β1, and further resulted in liver fibrosis [3,5]. In Figure 5a, ROS levels can be seen to be significantly increased by CCl_4_ i.p. injection. However, these test substances including red quinoa powder and its extracts suppressed CCl_4_-induced ROS levels. The HL-P group displayed the most significant effect among the other test substance groups. In order to investigate the regulation of proinflammatory factor expression, TNF-α and IL-6 expressions were determined by using ELISA kits. In Figure 5b,c after CCl_4_ i.p. injection (CCL group) had significantly higher levels of TNF-α and IL-6 expressions compared to the NOR group (*p* < 0.05). After treatment of the samples, TNF-α and IL-6 expressions in the HL-P, HL-E and Rutin group were inhibited significantly (*p* < 0.05). However, the HL-W group only showed significantly lower expression of TNF-α, compared to the CCL group (*p* < 0.05). After CCl_4_ i.p. injection, TGF-β1 expression in the CCL group was significantly higher than the NOR group (*p* < 0.05). The HL-P and Rutin groups had significantly lower TGF-β1 expression compared to the CCL group (*p* < 0.05). However, the HL-E and HL-W groups showed no difference to the CCL group (*p* > 0.05) regarding TGF-β1 expression. α-SMA expression indicated the activation of HSCs. The HL-W group had significantly lower α-SMA expression compared to the CCL group (*p* < 0.05) (as shown in Figure 6).

## 4. Discussion

Red quinoa gradually developed as a popular grains and dietary supplement due to its health functions. A previous study indicated quinoa seed (*Chenopodium* quinoa) showed a hepatoprotective effect against CCl_4_-induced liver damage. *Chenopodium* quinoa seed lowered CCl_4_-induced AST and ALT activities and liver damage by raising SOD and GSH levels [15]. However, the mechanisms associated with the protection against CCl_4_-induced inflammation and fibrosis were still unclear. Furthermore, red quinoa (*Chenopodium formosanum* Koidz), a native plant in Taiwan, was different to *Chenopodium* quinoa. Therefore, this study is the first study to investigate the effects of a novel grain red quinoa and its extracts on the prevention of CCl_4_-induced liver injury and fibrosis in mice. This study is an important study to develop red quinoa as a novel natural supplementary and functional food. Importantly, the previous study suggested that rutin can perform liver protection [12], but the studies of anti-liver fibrosis are still rare in the current literature. Red quinoa is rich in proteins, minerals, essential amino acids and dietary fibre. Previous studies also indicated that betacyanins, quercetin, rutin and other flavonoids can be found in the red quinoa. These compounds were also proven to perform antioxidative and anti-inflammatory effects [6,7,8]. Flavonoids were proven to decrease hepatic lipid accumulation, lipid peroxidation, oxidative stress and inflammation in the mouse model with alcohol-induced hepatic damage [16]. Therefore, these bioactive compounds should display liver protection. 

CCl_4_ is the most common hepatotoxin to be used to research liver injury and fibrosis [17]. Repeated and chronic CCl_4_ i.p. injection can result in liver enlargement [18]. According to the results, the CCl_4_ i.p. injection resulted in significantly higher levels of serum AST and ALT. H&E staining and Sirius red staining of the liver showed that CCl_4_ i.p. injection also resulted in inflammation, necrosis and collagen accumulation, and fibrosis in the liver tissue. Therefore, the CCl_4_ i.p. injection provided a significant animal model for liver injury. However, after treating the samples, the levels of serum AST and ALT were decreased significantly by red quinoa and its bran extracts. Histological assays also confirmed that treatment with red quinoa and its bran extracts ameliorated liver injury necrosis and inflammation. Furthermore, rutin always provided significant improvement regarding liver injury in the results, which suggested that it can be regarded as the functional compound of red quinoa bran. In the previous study, rutin also has been proven the protective effect on methotrexate-induced liver injury in rats [19].

CCl_4_ was proven to induce oxidative stress and lipid peroxidation in the liver [20]. Previous studies showed that chronic CCl_4_ i.p. injection results in significantly lower activities of SOD, CAT, GPx and GRd, a significantly lower level of GSH and a significantly higher level of thiobarbituric acid reactive substances [21,22]. In this study, CCl_4_ i.p. injection resulted in significantly higher levels of MDA and weaker activities of SOD and CAT. The activities of SOD and CAT and the levels of GSH were increased with red quinoa and its bran ethanol extract. The bran water extract performed weaker CAT and GSH raising effects than the bran ethanol extract. This was because a higher concentration of rutin in the bran can be extracted with ethanol rather than water. There are many studies showing that rutin enhances antioxidant defence systems against hepatic oxidative stress in animal models [23,24]. Rutin from *Fagopyrum tataricum* increased the activities of SOD, CAT, GPx and GRd in the ethanol liver injury in mouse and CCl_4_-induced liver injury in rats [25]. Betanin and isobetanin from red quinoa are also antioxidative agents [8]. Therefore, the antioxidative system activation is from the rutin and the other polyphenolic compounds of the red quinoa and its bran.

ROS and lipid peroxidation damage DNA in the cells resulted in inflammation. Macrophages release pro-inflammatory cytokines, such as TNF-α, IL-1β and IL-6, to enhance inflammatory responses, which then results in further liver injury [2]. In this study, CCl_4_ i.p. injection enhanced the expressions of TNF-α and IL-6. With the red quinoa and its bran ethanol extract treatments, the expressions of TNF-α and IL-6 were inhibited in mice. Rutin has shown a hepatoprotective effect in animals. In a cyclophosphamide-induced liver inflammation model, rutin downregulated the TNF-α/IL-6 and NFκB/MAPK pathways to protect the liver [10]. Rutin also ameliorated high-cholesterol diet-induced liver inflammation in rats through downregulating the TGF-β/Smad pathway in rats [26].

Because rutin was the major flavonoids compound in the red quinoa, this study investigated the effect and role of rutin on the regulation of antioxidative enzyme and pro-inflammatory factors expression. In this study, the results of catalase, SOD, ROS, TGF-β1 and collagen formation in red quinoa powder all performed more significant effect than rutin. Therefore, rutin was proven to be the functional compound, but red quinoa also contained other rich flavonoids. Therefore, red quinoa should provide more significant liver protection than rutin for blocking the development of liver fibrosis via suppressing TGF-β1 in this study.

## 5. Conclusions

**Figure 7:**
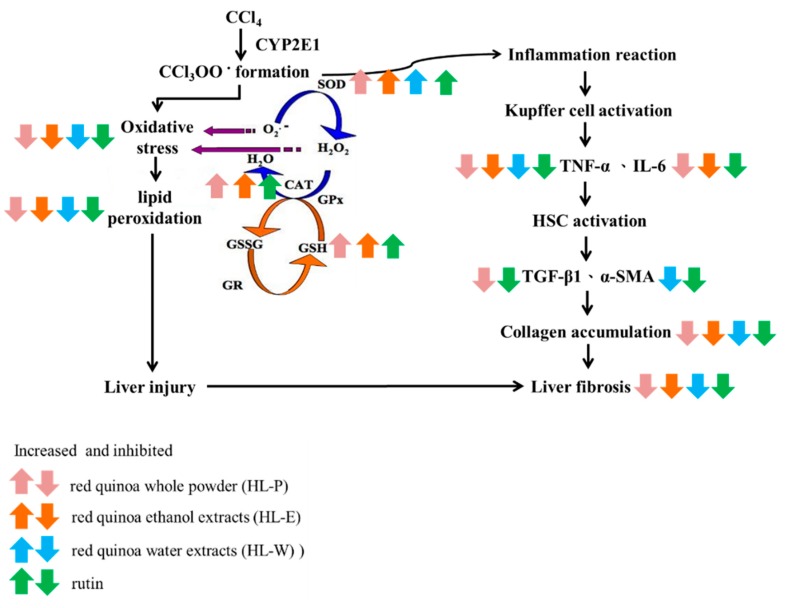
The regulation of red quinoa, red quinoa extracts and rutin in liver of CCl_4_-induced liver injury mice.

Chronic liver injury eventually causes liver fibrosis and cirrhosis. In this study, CCl_4_ i.p. injection not only raised AST and ALT activity but also significantly blocked the antioxidative systems by lowering SOD and CAT activity. Inflammatory response and collagen accumulation were found in the mice treated with CCl_4_ i.p. injection due to the expression of proinflammatory response factors TNF-α, IL-6 and TGF-β1. The result indicated that red quinoa, bran ethanol extract, bran water extract and rutin all significantly decreased CCl_4_ raised AST and ALT activity and collagen accumulation. Its mechanism was involved in the antioxidative system activation, suppressing the TNF-α/IL-6 pathway and blocking the TGF-β1 pathway. However, rutin in the bran ethanol extract may be one of the bioactive compounds to prevent CCl_4_-induced oxidative stress and liver inflammation because red quinoa powder had a greater effect than rutin. To summarize, rutin and rich antioxidative agents of the red bran quinoa can be extracted with ethanol, which can be developed as the novel functional food to ameliorate CCl_4_-induced oxidative stress, inflammation and fibrosis in the liver through enhancing anti-oxidative enzymes and suppressing the TNF-α/IL-6 pathway (Figure 7).

## Figures and Tables

**Table 1:** The effects of red quinoa and its extracts on the body weight gain of the CCl_4_-induced liver injury mice.

Groups	Body Weight (g)			
	0th Week	2nd Week	4th Week	6th Week
NOR	25.8 ± 1.4 ^a^	26.4 ± 0.5 ^b^	27.5 ± 1.8 ^b^	29.0 ± 0.9 ^b^
CCL	26.0 ± 1.5 ^a^	24.5 ± 0.8 ^a^	24.5 ± 3.4 ^a^	26.5 ± 1.9 ^a^
HL-P	25.5 ± 1.6 ^a^	25.0 ± 1.3 ^a^	24.6 ± 2.2 ^a^	26.5 ± 1.2 ^a^
HL-E	25.9 ± 1.5 ^a^	24.4 ± 1.1 ^a^	24.3 ± 2.8 ^a^	26.6 ± 1.6 ^a^
HL-W	25.6 ± 1.6 ^a^	24.8 ± 1.0 ^a^	24.2 ± 1.5 ^a^	26.0 ± 2.3 ^a^
Rutin	25.8 ± 1.5 ^a^	24.4 ± 2.1 ^a^	24.7 ± 3.3 ^a^	26.0 ± 2.3 ^a^

NOR: normal group, CCL: CCl_4_ induced liver injury mice, HL-P: CCl_4_ induced liver injury mice fed 5.13 g/kg (rutin 8.46 mg/kg) of red quinoa-powder, HL-E: CCl_4_ induced liver injury mice fed 1.54 g/kg (rutin 16.4 mg/kg) of red quinoa-ethanol extracts, HL-W: CCl_4_ induced liver injury mice fed 1.54 g/kg (rutin 3.92 mg/kg) of red quinoa-water extracts, Rutin: CCl_4_ induced liver injury mice fed 16.4 mg/kg of rutin. Data are presented as mean ± SD (*n* = 8). ^a,b^ Different letters indicate significantly different values according to a one-way ANOVA using Duncan’s multiple test (*p* < 0.05).

**Table 2:** The effects of red quinoa and its extracts on body weight, liver weight and liver weight/body weight ratio of the CCl_4_-induced liver injury mice.

Groups	Liver Weight (g)	Body Weight (g)	Liver Weight/Body Weight (%)
NOR	1.38 ± 0.10 ^a^	27.63 ± 1.41 ^d^	5.00 ± 0.15 ^a^
CCL	2.00 ± 0.18 ^cd^	25.50 ± 1.07 ^abc^	7.87 ± 0.75 ^cd^
HL-P	1.82 ± 0.16 ^bc^	24.63 ± 1.92 ^ab^	7.39 ± 0.36 ^bc^
HL-E	2.20 ± 0.28 ^d^	27.00 ± 2.20 ^d^	8.11 ± 0.61 ^de^
HL-W	2.20 ± 0.35 ^d^	25.75 ± 1.91 ^cd^	8.51 ± 0.90 ^e^
Rutin	1.65 ± 0.31 ^b^	23.38 ± 3.25 ^a^	7.04 ± 0.48 ^b^

NOR: normal group, CCL: CCl_4_ induced liver injury mice, HL-P: CCl_4_ induced liver injury mice fed 5.13 g/kg (rutin 8.46 mg/kg) of red quinoa-powder, HL-E: CCl_4_ induced liver injury mice fed 1.54 g/kg (rutin 16.4 mg/kg) of red quinoa-ethanol extracts, HL-W: CCl_4_ induced liver injury mice fed 1.54 g/kg (rutin 3.92 mg/kg) of red quinoa-water extracts, Rutin: CCl_4_ induced liver injury mice fed 16.4 mg/kg of rutin. Data are presented as mean ± SD (*n* = 8). ^a,b,c,d,e^ Different letters indicate significantly different values according to a one-way ANOVA using Duncan’s multiple test (*p* < 0.05).

**Table 3:** The effects of red quinoa and its extracts on serum AST, ALT and ALP activities of the CCl_4_-induced liver injury mice.

Groups	AST Activity (U/L)	ALT Activity (U/L)	ALP Activity (IU/L)
NOR	63.6 ± 4.2 ^a^	41.3 ± 3.2 ^a^	97.9 ± 10.0 ^c^
CCL	230.0 ± 69.8 ^c^	309.3 ± 31.6 ^d^	84.3 ± 4.8 ^b^
HL-P	95.8 ± 9.3 ^a^	215.0 ± 33.2 ^b^	84.9 ± 4.9 ^b^
HL-E	174.6 ± 45.5 ^b^	209.0 ± 36.9 ^b^	76.8 ± 2.8 ^a^
HL-W	173.9 ± 60.9 ^b^	226.8 ± 64.9 ^bc^	82.5 ± 6.6 ^ab^
Rutin	112.1 ± 19.6 ^a^	264.1 ± 71.0 ^c^	75.8 ± 6.9 ^a^

AST: aspartate aminotransferase, ALT: alanine transaminase, ALP: alkaline phosphatase, NOR: normal group, CCL: CCl_4_-induced liver injury mice, HL-P: CCl_4_-induced liver injury mice fed red quinoa-powder, HL-E: CCl_4_-induced liver injury mice fed red quinoa-ethanol extracts, HL-W: CCl_4_-induced liver injury mice fed red quinoa-water extracts, Rutin: CCl_4_-induced liver injury mice fed rutin. Data are presented as mean ± SD (*n* = 8). ^a,b,c,d^ Different letters indicate significantly different values according to a one-way ANOVA using Duncan’s multiple test (*p* < 0.05).

**Table 4:** The effects of red quinoa and its extracts on serum total protein, albumin, globulin, A/G ratio and total bilirubin of the CCl_4_-induced liver injury mice.

Groups	Total Protein (g/dL)	Albumin (g/dL)	Globulin (g/dL)	A/G Ratio	TBIL (mg/dL)
NOR	5.46 ± 0.28 ^a^	3.59 ± 0.16 ^a^	1.85 ± 0.11 ^a^	1.91 ± 0.06 ^a^	0.04 ± 0.01 ^a^
CCL	7.40 ± 0.80 ^c^	4.79 ± 0.22 ^c^	2.51 ± 0.50 ^c^	2.15 ± 0.17 ^b^	0.12 ± 0.02 ^d^
HL-P	6.59 ± 0.16 ^b^	4.38 ± 0.05 ^b^	2.20 ± 0.15 ^bc^	1.90 ± 0.11 ^a^	0.08 ± 0.01 ^b^
HL-E	6.64 ± 0.29 ^b^	4.43 ± 0.13 ^b^	2.25 ± 0.18 ^bc^	1.81 ± 0.08 ^a^	0.08 ± 0.01 ^b^
HL-W	6.81 ± 0.69 ^b^	4.46 ± 0.35 ^b^	2.35 ± 0.38 ^bc^	1.94 ± 0.26 ^a^	0.10 ± 0.03 ^cd^
Rutin	6.30 ± 0.52 ^b^	4.30 ± 0.25 ^b^	2.04 ± 0.21 ^ab^	1.83 ± 0.16 ^a^	0.09 ± 0.01 ^ab^

NOR: normal group, CCL: CCl_4_-induced liver injury mice, HL-P: CCl_4_ induced liver injury mice fed red quinoa-powder, HL-E: CCl_4_-induced liver injury mice fed red quinoa-ethanol extracts, HL-W: CCl_4_-induced liver injury mice fed red quinoa-water extracts, Rutin: CCl_4_-induced liver injury mice fed rutin. Data are presented as mean ± SD (*n* = 8). ^a,b,c,d^ Different letters indicate significantly different values according to a one-way ANOVA using Duncan’s multiple test (*p* < 0.05).

**Table 5:** The effects of red quinoa and its extracts on serum BUN and CRE of the CCl_4_-induced liver injury mice.

Groups	BUN (mg/dL)	CRE (mg/dL)
NOR	23.33 ± 2.13 ^a^	0.15 ± 0.02 ^a^
CCL	31.45 ± 5.39 ^b^	0.25 ± 0.05 ^d^
HL-P	26.54 ± 5.77 ^ab^	0.22 ± 0.06 ^cd^
HL-E	27.64 ± 4.02 ^ab^	0.19 ± 0.03 ^ab^
HL-W	33.99 ± 12.69 ^b^	0.21 ± 0.05 ^cd^
Rutin	32.85 ± 6.28 ^b^	0.21 ± 0.04 ^cd^

BUN: blood urea nitrogen, CRE: creatinine, NOR: normal group, CCL: CCl_4_-induced liver injury mice, HL-P: CCl_4_-induced liver injury mice fed red quinoa-powder, HL-E: CCl_4_-induced liver injury mice fed red quinoa-ethanol extracts, HL-W: CCl_4_-induced liver injury mice fed red quinoa-water extracts, Rutin: CCl_4_-induced liver injury mice fed rutin. Data are presented as mean ± SD (*n* = 8). ^a,b,c,d^ Different letters indicate significantly different values according to a one-way ANOVA using Duncan’s multiple test (*p* < 0.05).
